# DAILY STRESS, ANTICIPATORY ANXIETY, RUMINATION, AND NEGATIVE AFFECT IN LATE LIFE

**DOI:** 10.1093/geroni/igac059.1897

**Published:** 2022-12-20

**Authors:** Zexi Zhou, Karen Fingerman

**Affiliations:** The University of Texas at Austin, Austin, Texas, United States; The University of Texas at Austin, Austin, Texas, United States

## Abstract

Chronic and daily stress are risk factors to older adults’ health and well-being. However, most studies of stress have focused on the reactivity and recovery process following the onset of stress. Relatively little research has investigated the worries that precede stress (e.g., anticipatory anxiety) and how such worries may contribute to older adults’ subsequent emotional experiences and rumination, especially in daily settings. This study investigated the joint associations of daily stress and anticipatory anxiety on rumination and negative affect in older adults’ everyday life. We leveraged the ecological momentary assessment (EMA) data over 5 to 6 days from the Daily Experiences and Well-being Study (N = 267, Mage = 73.72). Anticipatory anxiety was moderately correlated with stresses experienced that day, r = .23, p < .001. We found significant joint associations between daily stress and anticipatory anxiety with rumination and negative affect. Higher daily anticipatory anxiety (M+1SD) combined with higher stress generated the highest rumination and negative affect, whereas lower daily anticipatory anxiety (M-1SD) paired with lower stress generated the lowest rumination and negative affect. Higher daily anticipatory anxiety paired with lower stress, or lower anticipatory anxiety paired with higher stress, led to a moderate level of rumination and negative affect. These results suggest that anticipatory anxiety toward potential stress has distinct negative effects on older adults’ daily experiences, and these effects may contribute to stressors and to heightened rumination. The findings highlight the role that proactive emotional expectations may play in older adults’ everyday life.

